# Cost-Effectiveness of Sacituzumab Govitecan Versus Chemotherapy in Metastatic Triple—Negative Breast Cancer in Taiwan

**DOI:** 10.3390/cancers17203305

**Published:** 2025-10-13

**Authors:** Shyh-Yau Wang, Yun-Sheng Tai, Henry W. C. Leung, Shin Hang Leung, Agnes L. F. Chan

**Affiliations:** 1 Department of Radiology, An-Nan Hospital, China Medical University, Tainan 709, Taiwan; 2 Department of Breast Surgery, An-Nan Hospital, China Medical University, Tainan 709, Taiwan; 073889@tool.caaumed.org.tw; 3 Department of Radiation Oncology, An-Nan Hospital, China Medical University, Tainan 709, Taiwan; 4 Department of Hospital and Health Care Administration (with Master’s Program), Chia Nan University of Pharmacy & Science University, Tainan 717301, Taiwan; 5 Department of Research, An-Nan Hospital, China Medical University, Tainan 709, Taiwan

**Keywords:** cost-effectiveness analysis, sacituzumab govitecan, single-agent chemotherapy, metastatic triple negative breast cancer

## Abstract

**Simple Summary:**

Sacituzumab govitecan (SG) is a novel antibody–drug conjugate (ADC) targeting trophoblast cell surface antigen 2 (TROP2) expressing cancer cells. It delivers the topoisomerase I inhibitor SN-38 to induce DNA damage-mediated apoptosis. Sacituzumab is approved for patients with unresectable, locally advanced or metastatic triple-negative breast cancer who has received two or more prior systemic therapies, at least one of which was for metastatic disease. Based on the positive results from the Phase III ASCENT clinical trial, sacituzumab offers an alternative new mechanism treatment option for patients with metastatic triple-negative breast cancer who have failed initial chemotherapy and have a poor prognosis. However, previous cost-effectiveness analyses conducted in the United States and China concluded that sacituzumab is not cost-effective for mTNBC at its current price, within acceptable willingness-to-pay thresholds in each country. The results of this study suggest that SG needs to lower its current price or increase the WTP threshold value in order to achieve cost-effectiveness.

**Abstract:**

Objective: This study evaluated the cost-effectiveness of sacituzumab govitecan (SG) compared with single-agent chemotherapy of the physician’s choice (TPC) from the perspective of Taiwan’s National Health Insurance. Methods: A partitioned survival model was developed to assess outcomes in patients with metastatic triple-negative breast cancer (mTNBC). Clinical data were derived from the ASCENT trial, while direct medical costs were obtained from Taiwan’s National Health Insurance Administration (NHIA). Utility values were taken from published literature. The primary outcome was the incremental cost-effectiveness ratio (ICER), expressed as cost per quality-adjusted life year (QALY) gained. One-way and probabilistic sensitivity analyses were performed to examine parameter uncertainty and test the robustness of the results. Results: In the base-case analysis, SG was associated with an incremental cost of USD 121,836 per QALY gained—exceeding Taiwan’s willingness-to-pay (WTP) threshold of USD 102,120. One-way sensitivity analyses indicated that SG drug cost was the primary driver of ICER variability. Probabilistic sensitivity analysis showed that reducing the price of SG by 50% increased the likelihood of cost-effectiveness. Conclusions: From the NHIA perspective, SG is not cost-effective for patients with advanced or metastatic TNBC at its current price. Substantial price reductions would be required for SG to become cost-effective under the WTP threshold of USD 102,120 per QALY.

## 1. Introduction

Triple-negative breast cancer (TNBC) is an aggressive subtype of breast cancer characterized by high mortality, early recurrence, and poor prognosis. Globally, it accounts for approximately 15–20% of all breast cancer cases [1]. Recent epidemiological studies have shown that the incidence of TNBC is rising among adolescents and young adults [2]. Women born after 1980 have a higher risk of developing breast cancer—particularly TNBC—compared with those born before 1950 [3]. In Taiwan, the incidence of TNBC increased from 15% in 2012 to 19% in 2022 [4].

Hormone receptor-positive, human epidermal growth factor receptor 2-negative (HR+/HER2−) breast cancer remains the predominant molecular subtype, representing about 70% of all breast cancer cases [5]. For these patients, endocrine therapy combined with cyclin-dependent kinase 4/6 (CDK4/6) inhibitors is the current standard first-line treatment [6]. Although CDK4/6 inhibitors are highly effective in treating HR+/HER2− breast cancer, their high cost often makes them less cost-effective in this setting. Previous cost-effectiveness studies have suggested lowering drug prices, potentially benefiting patients, and exploring their potential use in combination with other therapies or as monotherapy for triple-negative breast cancer (TNBC) to improve cost-effectiveness. However, CDK4/6 inhibitors are currently not considered cost-effective for treating TNBC because they are not approved for this specific subtype and are expensive [7,8]. TNBC management contributes disproportionately to healthcare expenditures: in the United States, it accounts for 5–10% of cancer costs and approximately 0.5% of the total healthcare budget [9]. In Taiwan, TNBC represented about 0.033% of total cancer treatment costs in 2022 [10]. These figures highlight both the clinical and economic burden of TNBC and the urgent need for effective and affordable therapies.

Sacituzumab govitecan (SG) is a novel antibody–drug conjugate (ADC) targeting trophoblast cell surface antigen 2 (TROP2) expressing cancer cells. It delivers SN-38, the topoisomerase I inhibitor, to induce DNA damage-mediated tumor cells apoptosis [11]. On 15 January 2024, Taiwan’s NHIA approved SG for reimbursement under restricted conditions: adult patients with unresectable locally advanced or metastatic TNBC who received at least two prior systemic therapies, including one for advanced disease. SG gained approval based on the phase 3 ASCENT trial, which demonstrated superior outcomes compared with standard chemotherapy, including significant improvements in progression-free survival (PFS), overall survival (OS), and objective response rate [12].

There is no doubt that SG offers a new treatment option for patients with metastatic triple-negative breast cancer (mTNBC). However, the cost of SG treatment is significantly higher, which may limit its popularity in many countries [13]. Currently, only a few economic evaluations of SG for the treatment of mTNBC have been conducted. Healthcare policymakers must assess cost-effectiveness to ensure efficient use of limited healthcare resources. Previous cost-effectiveness analyses conducted in the United States and China concluded that SG was not cost-effective for mTNBC at current prices [14,15,16,17,18]. Since SG has now been reimbursed in Taiwan, it is essential to evaluate whether its clinical benefits justify the economic burden. This study is the first to assess the cost-effectiveness of SG compared with chemotherapy for advanced or metastatic TNBC in Taiwan.

## 2. Methods

### 2.1. Model Structure

**Figure 1:**
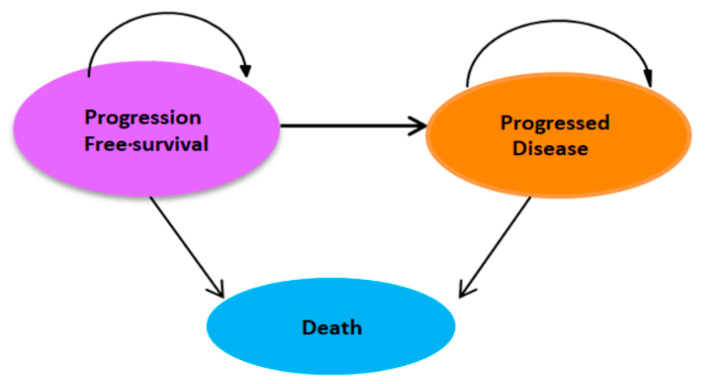
The partitioned survival model uses a three-state structure (progression-free, progressive disease, death) and arrows to illustrate transitions between these states.

A partitioned survival model (PSM) with three health states was developed to evaluate the costs and long-term outcomes of SG compared with TPC in patients with metastatic TNBC from the perspective of Taiwan’s NHIA. The three mutually exclusive health states were progression-free survival (PFS), progressed disease (PD), and death (Figure 1).

The proportion of patients in each health state at each cycle was estimated from OS and PFS curves reported in the ASCENT trial [12]. The area under the PFS curve represented the proportion of patients alive without progression, while the area under the OS curve represented total survival. A cycle length of one month was used, and the model adopted a 10-year time horizon to capture lifetime outcomes. Costs and health outcomes were discounted at an annual rate of 3% [19].

Primary outcomes included total costs, QALYs, ICERs, and net monetary benefit (NMB). NMB represents the total monetary value of an intervention by subtracting its costs from its benefits, all expressed in monetary terms, given a specific willingness-to-pay (WTP) threshold. A positive NMB suggests that the intervention is worthwhile compared to the alternatives at that threshold, offering a single, absolute measure of value rather than a ratio like the incremental cost-effectiveness ratio (ICER) [20]. The model was developed using TreeAge Pro version 2022 (TreeAge Software, Williamstown, MA, USA).

### 2.2. Clinical Data

**Figure A2:**
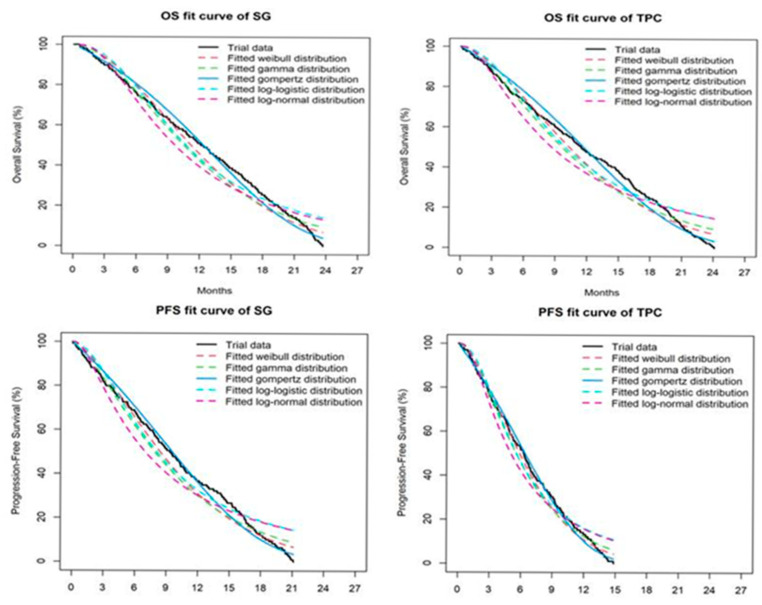
The model fitting results for Kaplan-Meier curve (B1) upper side: overall survival (OS) fit curve of SG (sacituzumab) and TPC (chemotherapy on physician ‘s choice); (B2) lower side: progression free (PFS) fit curve of SG and TPC.

As individual patient data from the ASCENT trial were unavailable, survival data were reconstructed from Kaplan–Meier curves using WebPlotDigitizer v4.6 and the Guyot algorithm [21]. Reconstructed curves are shown in Appendix A. The reconstructed curves were validated by comparing the model clinical outcomes with selected trial data (median OS and PFS). Based on the local oncologist’s assessment of clinical plausibility and goodness of fit, the goodness of fit of the reconstructed IPD with the standard parametric distributions of Weibull, Gamma, lognormal, and log-logistic was compared using the analytic Akaike distribution and the Bayesian information criterion [22,23]. Extrapolated curves informed long-term outcomes (Appendix B: Figure A2; Appendix C: Table A1).

### 2.3. Direct Medical Costs and Utility

The analysis adopted the NHIA perspective. Only Direct medical costs were included in this analysis (Appendix D Table A2). In the PFS state, drug costs included SG or TPC acquisition costs, administration costs, assessment costs (MRI costs), and the cost of treatment of grade 3/4 adverse events. In the PD state, costs included costs of MRI, palliative care or basic supportive care, and hospice per event (SAEs).

We investigated that the total drug costs of patients who received either SG at a dose of 10 mg per kilogram of body weight intravenously on days 1 and 8 of each 21-day cycle or single-agent chemotherapy or TPC (eribulin (1.4 mg/m^2^ IV, days 1 and 8 of a 21-day cycle), vinorelbine (25 mg/m^2^ IV, day 1 of a 7-day cycle), capecitabine (1000–1250 mg/m^2^ orally twice daily, days 1–14 of a 21-day cycle), or gemcitabine (800–1200 mg/m^2^ IV, days 1, 8, 15 of a 28-day cycle)) according to the ASCENT trial, To calculate the dosage of the above drugs, we adopted a mean body surface area (BSA) of 1.72 m^2^ and body weight of 57.3 kg, reflecting the Taiwanese female population [24]. Drug costs and other direct medical costs were collected based on the 2024 NHIA reimbursement list [25] and converted to US dollars (USD 1 = TWD 29) in June 2025. Only treatment costs of grade ≥ 3 SAEs were included, as lower-grade events incur minimal additional costs.

Appendix E Table A3 summarizes cost inputs. The willingness-to-pay (WTP) threshold was set at USD 102,120/QALY, which is 3 times the Taiwanese average monthly GDP per capita in 2024, to assess cost-effectiveness [26].

Utility scores for PFS and PD states ranged from 0 (death) to 1 (perfect health) and were derived from a previously published cost-effectiveness study [27,28]. We assumed 0.85 for SG in PFS, 0.58 for TPC in PFS, and 0.52 for PD. A disutility of 0.28 was applied for grade ≥ 3 adverse events, assumed to occur in the first cycle [27].

### 2.4. Base-Case Analysis

According to the recommendation of Guidelines for Pharmacoeconomic Evaluations in Taiwan [29], the incremental cost-effectiveness ratio (ICER) was calculated as the incremental cost per incremental quality adjusted life year (QALY) gained between the sacituzumab govitecan group and chemotherapy group. When the ICER was below the defined willingness-to-pay (WTP) threshold, the intervention was considered to be cost-effective. Costs and QALYs were discounted at an annual rate of 3% [19].

### 2.5. Sensitivity Analyses

One-way sensitivity analyses varied parameters by ±30% of baseline values (Appendix E: Table A3), with results presented in tornado diagrams [23,30]. Probabilistic sensitivity analysis (PSA) used 1000 Monte Carlo simulations, with gamma distributions for cost inputs and beta distributions for utilities. Cost-effectiveness acceptability curves compared SG and TPC against Taiwan’s WTP threshold.

### 2.6. Scenario Analyses

Three scenario analyses were conducted. The first was price reduction: reducing SG costs by 30% and 50%, while other direct medical costs remain unchanged in the progression-free survival state; the second is threshold analysis: raising the WTP threshold to a certain ratio in Taiwan, Netherlands, and England to compare the likelihood that SG treatment is cost-effective because these two countries adopt a universal public health insurance system similar to Taiwan, but with different policy support. The third was the BMN subgroup: The model for the brain metastasis negative (BMN) subgroup in the ASCENT trial was rerun to examine the cost-effectiveness of SG treatment.

## 3. Results

### 3.1. Internal Validity

**Figure 2:**
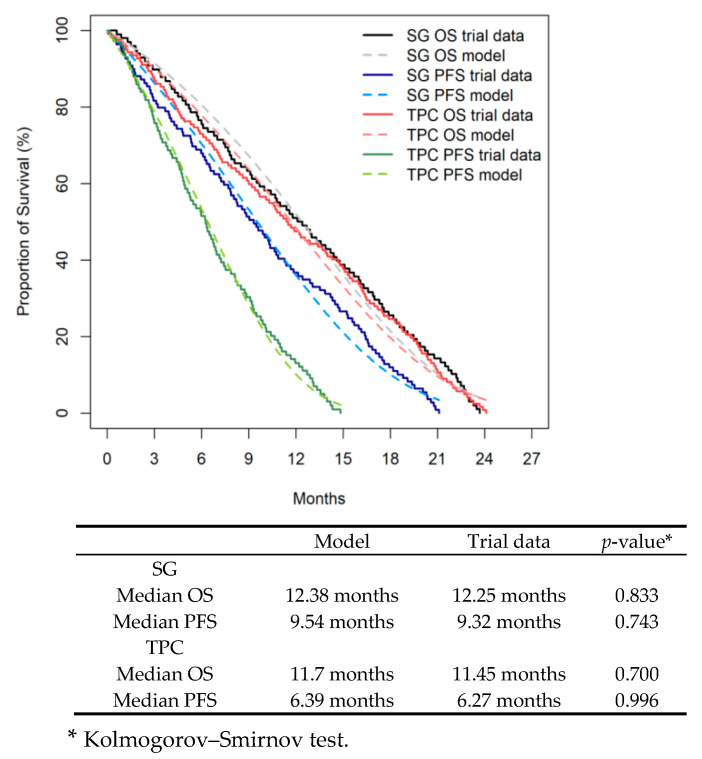
Internal validation of model (**Top**) Kaplan Meier OS and PFS curves from ACENT trial and model-generated for OS and PFS curves were shown. (**Bottom**) Table showed: modelled clinical outcomes were consistent with empirical data from the ACENT trial. SC, Sacituzumab, TPC, chemotherapy on physician’s choice.

The internal validation results of the reconstructed survival curves showed that the clinical outcomes predicted by the model were consistent with the empirical trial data in terms of median OS and PFS, confirming the internal validity of the parametric regression model (Figure 2).

### 3.2. Base-Case Analysis

SG provided 9.40 QALYs compared with 4.94 QALYs for TPC, yielding an incremental gain of 4.47 QALYs. The ICER was USD 113,277 per QALY gained. NMB was −111,074 for SG and USD 171,498 for TPC, indicating that SG was not cost-effective under Taiwan’s WTP threshold of USD 102,120/QALY, and a negative NMB represented that SG was not cost-effective compared with chemotherapy (Table 1).

### 3.3. One-Way Sensitivity Analyses

Tornado analyses showed that ICER estimates were most sensitive to SG cost in progression-free health state (PFS), and utility values for both SG and TPC in PFS and PD state also had substantial influence.

### 3.4. Probabilistic Sensitivity Analyses

PSA results showed that at the WTP threshold of USD 102,120/QALY, SG had a 29% probability of being cost-effective, compared with 71% for TPC.

### 3.5. Scenario Analyses

**Figure 3:**
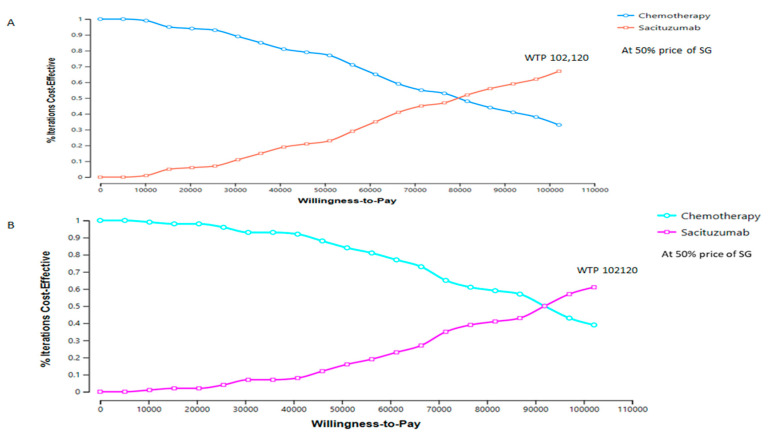
Cost-effectiveness acceptability curve for full population (**A**), no brain metastases subgroup. (**B**) at 50% price of sacituzumab govitecan (SG).

The first scenario results: Reducing SG’s price (cost) by 30% did not achieve cost-effectiveness. If the price of SG is reduced by 50%, SG has a 75% probability of being cost-effective, while TPC has only a 25% probability of being cost-effective (Table 2; Figure 3A). In both scenarios, the overall population ICER is within Taiwan’s WTP threshold (USD 102,120 per QALY gained).

Table 3 summarizes the results of the threshold analysis. In Taiwan, when the cost of sacituzumab was maintained at the baseline level and the willingness-to-pay (WTP) threshold was increased by 10% and 30%, sacituzumab demonstrated a 56% and 71% probability of being cost-effective, respectively, whereas treatment of the physician’s choice (TPC) showed corresponding probabilities of 44% and 29%. In the Netherlands and the United Kingdom, assuming the same sacituzumab cost as in Taiwan and adjusting the Netherlands’ WTP threshold to 10% of its maximum range (EUR 20,000–EUR 80,000 per quality-adjusted life year [QALY] gained), sacituzumab and TPC exhibited probabilities of cost-effectiveness of 87% and 13%, respectively. In contrast, in the United Kingdom, when the WTP threshold was raised to 2.9 times the current upper limit (GBP 20,000–GBP 30,000 per QALY gained), sacituzumab and TPC achieved probabilities of 57% and 43%, respectively. These differences may be attributed to the greater flexibility of WTP thresholds in the Netherlands and the United Kingdom, which vary according to disease severity and the potential to extend survival among terminally ill patients (often by several months) [31]. Similarly, WTP in Taiwan is a dynamic value that fluctuates with individual preferences, contextual factors, economic conditions, and demographic characteristics. Previous research in Taiwan has indicated that WTP for QALYs varies considerably depending on individual perspectives and uncertainties regarding future health states [32].

In the third scenario, i.e., the BMN subgroup, SG provided 2.6 incremental QALYs at an additional cost of USD 303,107. With a 50% price reduction, SG achieved a 70% probability of cost-effectiveness compared with 30% for TPC, which is consistent with the overall results (Figure 3B).

## 4. Discussion

SG has been reimbursed in Taiwan for nearly one year for patients with unresectable locally advanced or metastatic TNBC. While clinical outcomes are promising, our analysis indicates that SG is not cost-effective at current prices.

The cost of SG was the key driver of ICER outcomes, consistent with findings from U.S. and Chinese analyses [28,29]. Scenario analyses confirmed that only a 50% price reduction allowed SG to reach cost-effectiveness in both overall and BMN populations. These results highlight the significant economic challenges posed by innovative oncology drugs in Taiwan.

Our findings also align with recent analyses using data from other trials (TROPiCS-02, EVER-132-002), which similarly concluded that SG was not cost-effective across diverse populations [33,34,35,36]. Together, this suggests that SG’s market price may not reflect its clinical value.

In addition to the market price of sacituzumab, a major challenge in economic evaluation lies in defining an appropriate willingness-to-pay (WTP) threshold for each country. To date, there is no international consensus on how such thresholds should be determined. The World Health Organization (WHO) has recommended using one to three times the gross domestic product (GDP) per capita as a cost-effectiveness benchmark, particularly for low- and middle-income countries. However, few nations currently apply this guideline in practice. To illustrate cross-country variation, this study examined the United Kingdom and the Netherlands to explore the price levels at which sacituzumab would become cost-effective within their respective WTP thresholds. Both countries operate universal public health insurance systems similar to Taiwan’s, but their policy frameworks differ substantially. The Netherlands employs flexible thresholds ranging from EUR 20,000 to EUR 80,000 per quality-adjusted life year (QALY) gained, depending on disease severity. In contrast, the United Kingdom applies a narrower threshold of GBP 20,000–GBP 30,000 per QALY gained, typically for interventions that extend survival among terminally ill patients by approximately three months [31].

Our analysis suggests that, at Taiwan’s current sacituzumab price (USD 55,274 per course), cost-effectiveness could be achieved if the WTP thresholds were increased to 10% of the maximum range in the Netherlands and 30% in Taiwan. Conversely, the WTP threshold in the United Kingdom would need to be raised to 2.9 times its current upper limit for sacituzumab to be considered cost-effective. These results highlight the pivotal role of threshold selection in shaping economic conclusions—higher WTP thresholds substantially increase the probability of sacituzumab being cost-effective.

Comparable patterns can be observed across other high-income Asian economies with healthcare systems similar to Taiwan, including Japan, Singapore, and South Korea, as well as China, which is classified as an upper-middle-income economy [37]. All these countries implement universal public health insurance and assess cost-effectiveness using the incremental cost-effectiveness ratio (ICER) relative to a defined WTP threshold. When ICERs exceed the threshold, new technologies are typically considered less cost-effective, and price negotiations or adjustments are often pursued. For instance, South Korea’s WTP threshold is approximately KRW 30 million (around USD 22,000) per QALY for general cases and can increase to KRW 50 million for oncology drugs [38]. In Japan, several studies cite JPY 5 million (approximately USD 37,000) per QALY as a benchmark, with reported ranges extending up to JPY 15 million per QALY [39]. Singapore generally applies a WTP threshold equal to half its GDP per capita—around SGD 48,899 per QALY in 2021 [40]. Similarly, China adopts an ICER-based framework, with thresholds typically set at three times the GDP per capita, closely resembling Taiwan’s approach. Recent studies have reported China’s WTP ranging from USD 38,256 per QALY in 2022 to USD 38,201 per QALY in 2024 [14,28].

Reaching a consensus on the definition and application of WTP thresholds remains a longstanding yet critical issue in health economics. Standardized, transparent, and empirically grounded thresholds are essential to ensure that WTP estimates reflect collective social preferences and provide a robust foundation for policy decisions. Without such clarity, WTP estimates may be inconsistent or misleading, leading to inequitable or inefficient resource allocation. This underscores the importance of context-sensitive, evidence-based approaches to value measurement [41]. Despite decades of debate, achieving fairness and transparency in how healthcare resources are distributed among patients and across countries continues to be a key area for further research [42].

Another noteworthy finding of this study is the influence of health utility values as a sensitivity parameter. Because the ASCENT trial did not report utility data, prior cost-effectiveness analyses have relied on external estimates, which inevitably introduce uncertainty. Future research should therefore prioritize the inclusion of health utility assessments in phase III clinical trials or real-world quality-of-life studies to provide robust, locally relevant data and enhance the precision of cost-effectiveness analyses.

This study has several limitations. First, survival data were reconstructed from Kaplan–Meier curves using parametric extrapolation, which may introduce bias. Second, utility estimates were drawn from published studies rather than direct trial data. Third, assumptions regarding subsequent treatment strategies and best supportive care costs may also affect results. Nonetheless, the analysis provides important evidence for decision-making in Taiwan. Finally, this study may underscore the tension between clinical effectiveness and economic value in oncology. SG improves survival outcomes, but its high cost prevents it from being cost-effective under Taiwan’s WTP threshold.

## 5. Conclusions

At Taiwan’s current WTP threshold, SG is not a cost-effective treatment for advanced or metastatic TNBC. Substantial price reductions would be required for SG to achieve cost-effectiveness. Alternative reimbursement strategies, such as value-based pricing or pay-for-performance agreements [43,44], or adjusting the WTP threshold value depending on disease severity or patients’ age, may help balance innovation, affordability, and equitable patient access in the future.

## Figures and Tables

**Table 1:** Cost-effectiveness results in base-case analysis.

	Strategy	Cost	Increment Cost	Effectiveness	Incremental Effectiveness	ICER	NMB
Overall Population	Chemotherapy	75,273		4.94			171,498
	Sacituzumab	581,126	505,854	9.40	4.47	113,277	−111,074
BMN subgroup	Chemotherapy	75,273		4.94			171,498
	Sacituzumab	641,680	566,407	9.40	4.47	126,837	−171,627

Abbreviations: BMN, brain metastatic—negative; NMB, net monetary benefit; ICER, incremental cost-effectiveness ratio.

**Table 2:** Scenario analyses results of cost-effectiveness rankings at 30% and 50% price of SG.

Scenario 1	Strategy	Cost	Incr Cost	Effectiveness	Incr Eff	ICER	NMB
At 30% price of SG	Chemotherapy	53,738		3.5			120,873
Overall population	Sacituzumab	324,059	270,321	6.1	2.6	105,434	−21,254
BMN subgroup	Chemotherapy	53,738		3.5			120,873
	Sacituzumab	356,845	303,107	6.1	2.6	118,222	−54,039
**Scenario 2**	**Strategy**	**Cost**	**Incr Cost**	**Effectiveness**	**Incr Eff**	**ICER**	**NMB**
At 50% price of SG	Chemotherapy	53,738		3.5			120,873
Overall population	Sacituzumab	262,314	208,576	6.1	2.6	81,352	40,492
BMN subgroup	Chemotherapy	53,738		3.5			120,873
	Sacituzumab	285,247	231,509	6.1	2.6	90,296	17,559

Abbreviation: Incr, incremental; Eff, effectiveness; ICER, incremental cost-effectiveness ratio; NMB, net momentary benefit.

**Table 3:** Scenario of WTP threshold analysis.

	Scenario Description	Drug Cost (USD)		WTP Threshold	Probability of Cost-Effectiveness (%)	
1	In Taiwan, SG cost maintained at base-case value	SG	TPS	Cost/QALY	SG	TPC
	WTP threshold increased by 10%	55,274	6859	102,120	56	44
	WTP threshold increased by 30%	55,274	6859	112,330	71	29
2	In Netherlands, WTP threshold is EUR 20,000 to EUR 80,000 per QALY, assuming same drug cost as in Taiwan.					
	WTP threshold increased by 10% of USD 94,118	55,274	6859	103,530	52	48
3	In England, WTP threshold is GBP 20,000–GBP 30,000 per QALY, assuming the same drug cost as in Taiwan.					
	WTP threshold was raised to 2.9 times the current upper limit	55,274	6859	118,919 (GBP 88,000)	57	43

Annotation: 1 USD = EUR 0.85, (EUR 20,000–EUR 80,000 = USD 23,529–USD 94,118); 1 USD = 0.74 (GBP) British pound in Sept 2025. SG, Sacituzumab; TPC, chemotherapy of the physician’s choice.

**Figure A1:**
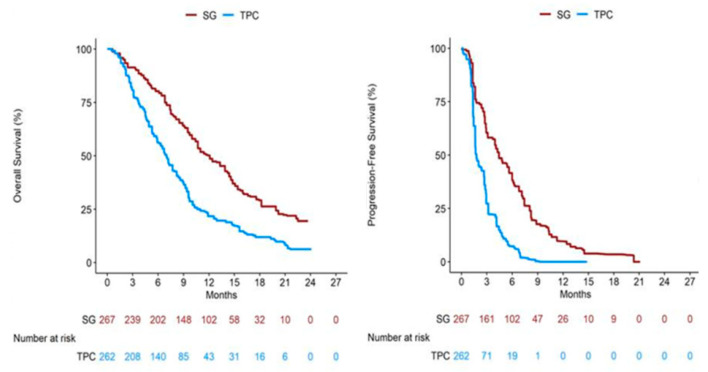
Reconstitution KM curves for overall (A1: left side) and progression-free survival (A2: right side).

## Data Availability

The data presented in this study are available on request from the corresponding author due to privacy.

## Appendix C

**Table A1:** Summary of estimated parameters and AIC/BIC values of PFS and OS models.

Strategies	Distributions	Parameters	OS						PFS					
			Est	L95%	U95%	SE	AIC	BIC	Est	L95%	U95%	SE	AIC	BIC
SG	Weibull	shape	1.792	1.518	2.116	0.152	654.085	659.255	1.474	1.258	1.726	0.119	700.750	706.133
		scale	13.749	12.247	15.434	0.811			10.775	9.433	12.308	0.731		
	Gompertz	shape	2.231	1.717	2.899	0.298	662.529	667.699	1.592	1.249	2.030	0.197	708.072	713.455
		rate	0.182	0.135	0.243	0.027			0.162	0.122	0.215	0.023		
	Gamma	shape	0.117	0.084	0.149	0.017	642.074	647.244	0.107	0.073	0.142	0.018	684.584	689.967
		rate	0.025	0.016	0.039	0.006			0.042	0.028	0.061	0.008		
	Log-logistic	shape	2.263	1.917	2.671	0.191	681.175	686.345	1.850	1.578	2.168	0.150	733.250	738.633
		scale	10.734	9.217	12.502	0.835			8.091	6.793	9.637	0.722		
	Log-normal	meanlog	2.268	2.109	2.428	0.081	684.600	689.770	1.941	1.745	2.137	0.100	745.931	751.314
		sdlog	0.807	0.701	0.928	0.058			1.044	0.915	1.193	0.071		
TPC	Weibull	shape	1.588	1.367	1.845	0.122	819.397	825.005	1.628	1.385	1.913	0.134	547.543	552.733
		scale	13.002	11.570	14.610	0.774			7.351	6.475	8.346	0.476		
	Gamma	shape	1.770	1.405	2.231	0.209	829.684	835.292	1.989	1.536	2.576	0.262	553.310	558.500
		rate	0.150	0.115	0.196	0.020			0.301	0.224	0.404	0.045		
	Log-logistic	shape	0.107	0.078	0.136	0.015	798.914	804.522	0.161	0.109	0.214	0.027	541.211	546.401
		rate	0.030	0.020	0.044	0.006			0.062	0.042	0.092	0.013		
	Gompertz	shape	1.978	1.703	2.296	0.151	856.600	862.208	2.137	1.813	2.520	0.180	570.547	575.737
		scale	9.951	8.523	11.618	0.787			5.569	4.745	6.537	0.455		
	Log-normal	meanlog	2.158	1.984	2.332	0.089	872.286	877.894	1.617	1.446	1.787	0.087	576.399	581.589
		sdlog	0.982	0.866	1.113	0.063			0.865	0.753	0.995	0.061		

## Appendix D

**Table A2:** Direct medical cost for each state in the model.

Parameters	Unit Cost (USD)		Total Cost (USD)		Source
	Sacituzumab	Chemotherapy	Sacituzumab	Chemo therapy	
**Progression free survival state, total cost**			66,340	9600	
*Drug Cost*	180 mg/vial		55,274	6859	TNHIA reimbursement price list [45]
Eribulin, 1.0 mg/2 mL/vial		297/vial		4336	
Vinorelbine 50 mg/5 mL/vial		208/vial		1248	
Capecitabine 500 mg/capsule		2.5/tablet		599	
Gemcitabine 1000 mg/vial		61.5/vial		676	
*Administration cost, total*			263	92	TNHIA reimbursement price list [46]
Physician OPD per visit	15.9	15.9	229	81	
Dispensing fee, per patient	2.38	2.38	34	11	
Laboratory test, total cost			115.8	35.2	
Complete blood count	6.897	6.897	38.6	11.7	
CA-125	13.79	13.79	77.2	23.5	TNHIA reimbursement price list [46]
*Assessment cost, total*			5796	1165	TNHIA reimbursement price list [46]
MRI with contrast	1554	1554	5796	1165	
*Managing grade 3/4 adverse events cost, total*			4891	1448	TNHIA reimbursement price list [47]
Leukopenia	382	382	2138	649	
Anemia	205	205	1153	349	
Neutropenia	288	288	1600	450	
**Progression disease health state, total cost**			18,337	7497	
MRI with contrast	1554				TNHIA reimbursement price list [46]
Palliative care	1206				[47]
Hospice per event.	1650				[48]

Abbreviations: OPD, out-patient department; MRI, magnetic resonance imaging. All cost were based on per patient per month were calculated in NTD. (New Taiwan Dollar, IUSD = NTD29) in July, 2025.

## Appendix E

**Table A3:** Model parameters varied in sensitivity analysis (±30%).

Variables	Baseline	Lower Range	Upper Range	Distribution	Reference
cPFS state	52,263	36,586	67,945	Gamma	TNHIA reference price list
cPFS1 state	9600	6720	12,480	Gamma	TNHIA reference price list
cPostProgression state	18,337	12,836	23,838	Gamma	TNHIA reference price list
cPostProgression state1	7497	5248	9746	Gamma	TNHIA reference price list
Rate OS	0.502	0.351	0.653	Beta	Restructed KM data
Rate PFS	0.473	0.331	0.615	Beta	Restructed KM data
Shape OS	0.117	0.082	0.152	Beta	Restructed KMdata
Shape PFS	0.107	0.075	0.139	Beta	Restructed KMdata
Utility PFS	0.907	0.635	0.635	Beta	[26]
Utility PP	0.730	0.511	0.949	Beta	[26]
Rate OS1	0.475	0.333	0.618	Beta	Restructed KMdata
Rate PFS1	0.505	0.353	0.657	Beta	Restructed KMdata
Shape OS1	0.107	0.075	0.139	Beta	Restructed KMdata
Shape PFS1	0.161	0.113	0.209	Beta	Restructed KMdata
Utility PFS1	0.860	0.602	1.118	Beta	[26]
Utility PP1	0.730	0.511	0.949	Beta	[26]
Time Horizon	10	3	13	Beta	

Annotation: cPFS, total cost in progression-free state of Sacituzumab govitecan (SG) group; cPFS1, total cost in progression free state of chemotherapy group (detailed data referred to both groups in Table 1); OS, overall survival of SG group; OS1, overall survival for chemotherapy; PP, progression state.
